# Predicting Severe COVID-19 Outcomes in the Elderly: The Role of Systemic Immune Inflammation, Liver Function Tests, and Neutrophil-to-Lymphocyte Ratio

**DOI:** 10.3390/healthcare12232429

**Published:** 2024-12-03

**Authors:** Adrian Vasile Bota, Felicia Marc, Mavrea Adelina, Laura Nicolescu, Adelina Georgiana Tudora, Coralia Cotoraci

**Affiliations:** 1 Doctoral School, Faculty of Medicine, “Vasile Goldis” Western University, Bulevardul Revolutiei 94, 310025 Arad, Romania; bota.adrian1@yahoo.com (A.V.B.); laura_dsp@yahoo.com (L.N.); adelina.tudora@yahoo.com (A.G.T.); 2 Department of Medical Sciences, Faculty of Medicine and Pharmacy, University of Oradea, 410073 Oradea, Romania; 3 Department of Internal Medicine I, Cardiology Clinic, “Victor Babes” University of Medicine and Pharmacy Timisoara, Eftimie Murgu Square 2, 300041 Timisoara, Romania; mavrea.adelina@umft.ro; 4 Department of Hematology, Faculty of Medicine, “Vasile Goldis” Western University, Bulevardul Revolutiei 94, 310025 Arad, Romania; rector_vg@uvvg.ro

**Keywords:** COVID-19, mortality, intensive care, SARS-CoV-2, prediction

## Abstract

**Background**: Patients aged 80 years and above are at increased risk for severe COVID-19 outcomes. This study aimed to evaluate the prognostic utility of the derived neutrophil-to-lymphocyte ratio (dNLR), aspartate-aminotransferase-to-lymphocyte ratio index (ALRI), aspartate-aminotransferase-to-platelet ratio index (APRI), and systemic immune inflammation index (SII) in predicting severe disease, intensive care unit (ICU) admission, and mortality among COVID-19 patients aged 80 years and older. **Methods**: In this retrospective cohort study, 138 elderly patients (≥80 years) and 215 younger controls (<65 years) with confirmed COVID-19 were included. Laboratory data at admission were collected, and the dNLR, ALRI, APRI, and SII scores were calculated. Receiver operating characteristic (ROC) curve analysis was performed to assess the predictive performance of these indices. **Results**: The SII had the highest area under the ROC curve (AUC) for predicting severe disease in elderly patients (AUC = 0.857, 95% CI: 0.795–0.919, *p* < 0.001), with an optimal cutoff value of 920 × 10⁹/L (sensitivity 86%, specificity 78%). Elevated SII was significantly associated with increased risk of ICU admission (hazard ratio (HR): 2.9, 95% CI: 1.8–4.6, *p* < 0.001) and mortality (HR: 3.2, 95% CI: 1.9–5.2, *p* < 0.001). Similarly, dNLR showed good predictive value (AUC = 0.792, 95% CI: 0.722–0.862, *p* < 0.001). **Conclusions**: SII and dNLR are valuable prognostic biomarkers for predicting severe outcomes in COVID-19 patients aged 80 years and above. Early identification using these indices can assist clinicians in risk stratification and management decisions to improve patient outcomes.

## 1. Introduction

The coronavirus disease 2019 (COVID-19) pandemic, caused by the severe acute respiratory syndrome coronavirus 2 (SARS-CoV-2), has posed unprecedented challenges to global health systems. While COVID-19 can affect individuals of all ages, the elderly population, particularly those aged 80 years and above, are disproportionately impacted, experiencing higher rates of hospitalization, severe disease, and mortality [1,2]. Additionally, patients with one or more comorbidities and those not vaccinated against COVID-19 are at a greater risk of developing severe forms of the disease [3]. Understanding the factors that contribute to adverse outcomes in this age group is crucial for improving patient care and allocating medical resources effectively.

Furthermore, age remains the most significant risk factor for severe COVID-19 outcomes, with the risk escalating markedly as age increases. Analysis of data published by the National Vital Statistics System (NVSS) at the National Center for Health Statistics (NCHS) demonstrates that compared to individuals aged 18–29 years, the risk of COVID-19 infection, hospitalization, and death is 25 times higher for those aged 50–64 years, 60 times higher for ages 65–74, 140 times higher for ages 75–84, and an astounding 340 times higher for those over 85 years old [4]. Additionally, data from our region highlight a strong correlation between the presence of comorbidities and negative COVID-19 outcomes. Conditions such as pre-existing lung diseases, cardiovascular diseases, neurological disorders, kidney diseases, hematological disorders, and metabolic syndrome are significantly associated with increased severity and mortality in COVID-19 patients [5,6]. These findings emphasize the critical need to prioritize age and comorbidities in risk assessment and management strategies to effectively mitigate the adverse impacts of COVID-19 on the most vulnerable populations.

The immune response in elderly patients is characterized by immunosenescence and a pro-inflammatory state known as inflammaging, which may influence disease progression and prognosis in COVID-19 [7,8]. Conventional prognostic tools may not fully capture the unique pathophysiological changes in elderly patients. Therefore, there is an urgent need to identify reliable and easily accessible biomarkers that can predict severe outcomes in this vulnerable population.

Hematological indices derived from routine laboratory tests—such as the derived neutrophil-to-lymphocyte ratio (dNLR), aspartate-aminotransferase-to-lymphocyte ratio index (ALRI), aspartate-aminotransferase-to-platelet ratio index (APRI), and systemic immune inflammation index (SII)—have emerged as potential prognostic markers in various diseases, including COVID-19 [9,10,11,12]. These indices reflect the balance between immune and inflammatory responses and have the advantage of being cost-effective and readily available in clinical settings.

Previous studies have demonstrated the prognostic value of these indices in predicting disease severity and mortality in COVID-19 patients [13,14,15,16]. However, most of these studies have focused on the general population or specific subgroups, with limited data on their applicability in patients aged 80 years and above. Given the unique physiological characteristics of the elderly, it is essential to assess whether these indices maintain their predictive value in this age group.

This study aims to evaluate the prognostic utility of dNLR, ALRI, APRI, and SII in predicting severe disease, ICU admission, and mortality among COVID-19 patients aged 80 years and older. By comparing these findings with a control group of patients younger than 65 years, we seek to determine the effectiveness of these indices in the elderly population and provide insights that could enhance risk stratification and management strategies for this high-risk group.

## 2. Materials and Methods

### 2.1. Legal and Ethical Considerations

The study was conducted at the Department of Clinical Infectious Diseases for Adults of the Clinical Emergency Hospital of Arad, in collaboration with the Victor Babes Hospital of Infectious Diseases Timisoara, affiliated with the Victor Babes University of Medicine and Pharmacy, a tertiary care hospital, from January 2022 to June 2024. A control group of patients under 65 years old was included for comparative analysis. The study protocol was approved by the Institutional Review Board, and written informed consent was obtained from all participants or their legal guardians. The study adhered to the ethical standards of the Declaration of Helsinki, EU Good Clinical Practice Directives (2005/28/EC), and the International Council for Harmonization of Technical Requirements for Pharmaceuticals for Human Use (ICH) guidelines. All patient data were anonymized to ensure confidentiality.

PICO statement: (1) population: patients aged 80 years and above with confirmed COVID-19 (elderly group) and patients younger than 65 years with confirmed COVID-19 (control group); (2) intervention: not applicable (observational study); (3) comparison: comparison between elderly patients (≥80 years) and younger patients (<65 years); (4) outcome: prognostic utility of dNLR, ALRI, APRI, and SII in predicting severe disease, ICU admission, and mortality.

### 2.2. Inclusion and Exclusion Criteria

To be eligible for inclusion in this study, participants in the elderly group must meet the following criteria: (1) they must be aged 80 years or older; (2) they must have a laboratory-confirmed diagnosis of COVID-19, verified through RT-PCR testing; and (3) they must have comprehensive laboratory data available at the time of admission, necessary for the calculation of the hematological indices under investigation (dNLR, ALRI, APRI, and SII).

For the control group, the inclusion criteria include: (1) an age less than 65 years; (2) a laboratory-confirmed diagnosis of COVID-19 through RT-PCR; and (3) the availability of complete laboratory data at admission.

The exclusion criteria, which apply universally across both groups, include: (1) individuals aged between 65 and 79 years to clearly differentiate between the elderly and younger cohorts; (2) any missing laboratory data necessary for calculating the specified indices; (3) the presence of chronic liver disease, any active hematological disorders, or active malignancy, as these conditions could independently influence the hematological indices and potentially confound the outcomes; and (4) current use of any immunosuppressive therapy, which could alter immune response and inflammatory markers, affecting the validity of the indices as prognostic tools.

### 2.3. Study Variables and Definitions

The primary outcomes of this study include severe COVID-19 disease, which is defined according to the World Health Organization (WHO) criteria, ICU admissions, and in-hospital mortality [17]. These outcomes are pivotal for evaluating the severity and immediate impact of COVID-19 in hospitalized patients. Additionally, secondary outcomes focus on the length of hospital stay and the need for mechanical ventilation, providing further insights into the extended effects and resource utilization associated with managing severe cases of COVID-19. These metrics collectively aid in assessing the comprehensive burden of the disease and the healthcare needs of infected individuals.

Data were extracted from electronic medical records, including demographic information (age, sex), comorbidities assessed by the Charlson Comorbidity Index (CCI), clinical presentations, laboratory results at admission, and clinical outcomes. Laboratory parameters collected included complete blood count (CBC), liver function tests (AST, ALT), and other relevant biochemical tests. All laboratory tests were collected at admission to the Infectious Disease Clinic.

1. Derived neutrophil-to-lymphocyte ratio (dNLR): calculated as Neutrophil Count/(Total Leukocyte Count − Neutrophil Count) [18].
2. Aspartate-aminotransferase-to-lymphocyte ratio index (ALRI): calculated as AST level (U/L)/Lymphocyte Count (×10⁹/L) [19].
3. Aspartate-aminotransferase-to-platelet ratio index (APRI): calculated as [(AST level/Upper Limit of Normal AST)/Platelet Count (×10⁹/L)] × 100 [20]. The upper limit of normal (ULN) for AST was considered 40 U/L.
4. Systemic immune inflammation index (SII): calculated as (Platelet Count × Neutrophil Count)/Lymphocyte Count [21].

### 2.4. Statistical Analysis

Statistical analyses were performed using SPSS version 26.0 (IBM Corp., Armonk, NY, USA). Continuous variables were presented as mean ± standard deviation (SD) or median with interquartile range (IQR) as appropriate. Categorical variables were expressed as frequencies and percentages. Comparisons between groups were made using the Student’s *t*-test or Mann–Whitney U test for continuous variables and the –hi-square test or Fisher’s exact test for categorical variables. Receiver operating characteristic (ROC) curve analysis was used to assess the predictive performance of dNLR, ALRI, APRI, and SII for severe disease, ICU admission, and mortality. The area under the ROC curve (AUC) was calculated, and optimal cutoff values were determined using the Youden index. Kaplan–Meier survival analysis was performed to compare survival between groups stratified by the optimal cutoff values of the indices. Differences in survival were assessed using the log-rank test. Cox proportional hazards regression was used to identify independent predictors of mortality, adjusting for potential confounders. A *p*-value < 0.05 was considered statistically significant.

## 3. Results

A total of 138 elderly patients (≥80 years) and 215 control patients (<65 years) met the inclusion criteria and were included in the analysis. The mean age of the elderly group was 83.7 ± 3.1 years, and the control group had a mean age of 52.5 ± 9.2 years. There was no significant difference in gender distribution between the groups (female: 80 (58%) elderly vs. 122 (57%) control, *p* = 0.81). Elderly patients had higher CCI scores (mean CCI: 4.4 ± 1.3 vs. 2.0 ± 0.8, *p* < 0.001), indicating a greater burden of comorbidities. The most common comorbidities in the elderly group were hypertension (98 (71%)) and diabetes mellitus (62 (45%)). The severity of COVID-19 was matched between the two groups, therefore there was no significant difference between groups in terms of clinical severity. Table 1 summarizes the demographic and clinical characteristics.

At admission, elderly patients had significantly higher neutrophil counts (7.9 ± 2.6 × 10⁹/L vs. 5.5 ± 2.2 × 10⁹/L, *p* < 0.001) and lower lymphocyte counts (0.9 ± 0.4 × 10⁹/L vs. 1.5 ± 0.5 × 10⁹/L, *p* < 0.001) compared to controls. AST levels were elevated in the elderly group (56 ± 21 U/L vs. 38 ± 14 U/L, *p* < 0.001), and platelet counts were lower (178 ± 52 × 10⁹/L vs. 222 ± 64 × 10⁹/L, *p* < 0.001). The calculated indices dNLR, ALRI, APRI, and SII were significantly higher in the elderly group (*p* < 0.001). Table 2 presents the laboratory findings and indices.

ROC curve analysis demonstrated that SII had the highest area under the curve (AUC) for predicting severe disease in elderly patients (AUC = 0.857, 95% CI: 0.795–0.919, *p* < 0.001). The optimal cutoff value for SII was 920 × 10⁹/L, yielding a sensitivity of 86% and specificity of 78%. dNLR also showed good predictive value (AUC = 0.792, 95% CI: 0.722–0.862, *p* < 0.001) with an optimal cutoff of 1.1 (sensitivity 80%, specificity 73%). ALRI and APRI had lower AUCs but remained significant predictors. Table 3 summarizes these findings.

ROC curve analysis for predicting ICU admission in elderly COVID-19 patients demonstrated that the systemic immune inflammation index (SII) exhibited the highest predictive accuracy with an area under the curve (AUC) of 0.869 (95% CI: 0.801–0.934, *p* < 0.001). The optimal cutoff value for SII was determined to be 966 × 10^9^/L, achieving a sensitivity of 88% and a specificity of 81%, indicating strong discriminative power for identifying patients at risk of ICU admission. Similarly, the derived neutrophil-to-lymphocyte ratio (dNLR) also showed good predictive value with an AUC of 0.805 (95% CI: 0.734–0.876, *p* < 0.001), and a cutoff value of 3.2 yielded a sensitivity of 83% and a specificity of 76%.

The aspartate-aminotransferase-to-lymphocyte ratio index (ALRI) and aspartate-aminotransferase-to-platelet ratio index (APRI) demonstrated lower, yet significant, predictive capabilities with AUCs of 0.778 (95% CI: 0.708–0.840, *p* < 0.001) and 0.754 (95% CI: 0.681–0.827, *p* < 0.001), respectively. For ALRI, a cutoff of 53 showed a sensitivity of 79% and a specificity of 70%, while APRI with a cutoff of 0.74 had a sensitivity of 75% and a specificity of 67% (Table 4).

Cox proportional hazards regression identified SII above 920 × 10⁹/L as an independent predictor of mortality in elderly patients (Hazard Ratio [HR]: 3.2, 95% CI: 1.9–5.2, *p* < 0.001), after adjusting for age, gender, and comorbidities. Elevated dNLR was also an independent predictor (HR: 2.4, 95% CI: 1.5–3.8, *p* = 0.001). ALRI and APRI were not significant predictors in the multivariate model (Table 5). In the control group, the indices were also associated with severe disease and mortality, but the predictive values were lower compared to the elderly group. For instance, SII had an AUC of 0.762 (95% CI: 0.691–0.833, *p* < 0.001) in the control group versus 0.857 in the elderly group.

**Figure 1:**
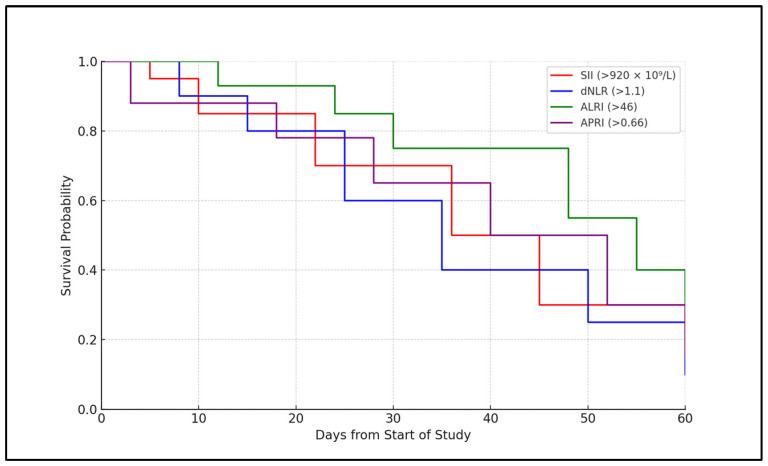
Kaplan–Meier analysis for mortality.

Kaplan–Meier survival curves were plotted for elderly patients stratified by the optimal cutoff values of SII and dNLR. Patients with SII above 920 × 10⁹/L had significantly lower survival probability compared to those below this value (31% vs. 81% survival at Day 30, log-rank *p* < 0.001). Similarly, elevated dNLR was associated with reduced survival (log-rank *p* < 0.001). Figure 1 illustrates the survival curves.

## 4. Discussion

### 4.1. Analysis of Findings

This study evaluated the prognostic utility of dNLR, ALRI, APRI, and SII in COVID-19 patients aged 80 years and above. The choice to analyze these parameters was rooted in their ability to reflect systemic inflammation and immune response, which are crucial in the pathophysiology of COVID-19. By contrasting these findings between the two age groups, the study aimed to validate the effectiveness of these indices specifically in elderly patients, who are inherently prone to severe complications due to diminished physiological reserves and higher comorbidity rates. The insights derived from this comparative analysis were expected to significantly enhance risk stratification, which could guide more tailored and effective management strategies for the elderly, ultimately aiming to improve their clinical outcomes.

The findings indicate that elevated SII and dNLR at admission are significant predictors of severe disease, ICU admission, and mortality in this age group. SII demonstrated the highest predictive value, with excellent sensitivity and specificity. The superior performance of SII may be attributed to its comprehensive reflection of the immune and inflammatory responses, integrating neutrophil, lymphocyte, and platelet counts [22]. Neutrophils play a critical role in the inflammatory response, while lymphopenia has been associated with impaired immune function in COVID-19 [23]. Platelets are involved in coagulation and immune regulation, and thrombocytopenia has been linked to worse outcomes [24]. Therefore, SII captures the complex interplay of these components, making it a robust prognostic marker.

The elevated dNLR also showed good predictive value. It is a simpler index that reflects the balance between neutrophil-mediated inflammation and lymphocyte-mediated immune response [25]. The findings suggest that both SII and dNLR can be useful tools for early risk stratification in elderly COVID-19 patients. The ALRI and APRI indices, which include AST levels, were less predictive in our study. This may be due to the multifactorial causes of elevated AST in elderly patients, including medications, comorbidities, and age-related hepatic changes, which may reduce their specificity for COVID-19 severity [26]. Compared to the control group, the predictive values of these indices were higher in the elderly patients. This suggests that these biomarkers may be particularly useful in the elderly population, who are at greater risk of severe outcomes.

In a similar manner, the study by Xiao-Yu Zhang et al. [27] found that specific hematological markers could predict severe or critical COVID-19 outcomes in older adults. Specifically, they identified CD4 cells and D-dimer as independent risk factors, with cut-off values determined at 268 cells/µL for CD4 cells and 0.65 mg/L for D-dimer. The combination of these predictors reached an AUC of 0.812, demonstrating moderate predictive efficiency for severe or critical illness in this population. Similarly, Zhihua Yu et al. [28] explored the clinical outcomes of COVID-19 in patients aged ≥75 years and identified elevated markers such as D-dimer, along with clinical symptoms like fever and low oxygen saturation (SpO2 ≤ 90%), as significant predictors of in-hospital death. The study showed that male sex, body temperature > 37.3 °C, and NT-proBNP levels > 1800 ng/L were independent risk factors for mortality, presenting an odds ratio as high as 273.5 for NT-proBNP.

Moreover, the study by Wassan Nori [29] highlighted the significant role of C-reactive protein (CRP) as a diagnostic and prognostic biomarker in assessing the risk of mortality among elderly COVID-19 patients. CRP, an acute-phase protein, was emphasized for its utility in following up and potentially targeting therapeutically to mitigate the severe effects of the virus in older adults. This reflects a focused approach on inflammatory markers as crucial indicators of disease severity and outcome, akin to findings from Anita Pirabe et al. [30], who investigated the relationship between monocyte activation markers and cytokine patterns, including interleukin 6 (IL-6), IL-8, and tumor necrosis factor (TNF), in elderly COVID-19 patients. Pirabe’s research did not find age-associated differences in monocyte counts but did find that adverse outcomes in older patients were correlated with a distinct cytokine expression pattern over the course of hospitalization, suggesting an age-driven inappropriate immune response.

In a similar manner, the study by Grigoras et al. [31] identified specific liver function tests and fibrosis scores as significant predictors of mortality in elderly COVID-19 patients. Notably, they found that liver biomarkers such as ALP, LDH, AST, and ALT were significantly associated with increased in-hospital mortality, with odds ratios ranging from 1.26 for ALP to 2.34 for ALT. Furthermore, higher scores on liver fibrosis indicators like APRI (>1.5), NFS (>1.5), and FIB-4 (>3.25) were correlated with increased mortality risks—2.69, 3.05, and 3.13 times, respectively. This underscores the critical role of liver-related parameters in predicting severe outcomes in geriatric patients with COVID-19. Similarly, the study by Fabiola Olivieri et al. [32] explored the predictive value of routine laboratory parameters, including complete blood count, in determining in-hospital mortality among geriatric COVID-19 patients. They highlighted that parameters such as neutrophilia, eosinopenia, lymphopenia, and neutrophil-to-lymphocyte ratio (NLR) had the highest predictive values for mortality.

Additionally, the integration of these hematological indices into existing clinical protocols could significantly enhance the early identification and management of high-risk elderly COVID-19 patients. Implementing routine screening for SII and dNLR upon hospital admission can facilitate prompt decision-making regarding the necessity for intensive monitoring and aggressive therapeutic interventions. Moreover, combining these biomarkers with other clinical parameters, such as vital signs and imaging findings, may improve the overall accuracy of prognostic models, leading to more personalized and effective patient care strategies [33,34,35]. Future research should focus on validating these findings in larger, multi-center cohorts and exploring the synergistic effects of combining multiple biomarkers to refine risk stratification further. Additionally, investigating the dynamic changes in these indices throughout the course of the illness could provide deeper insights into disease progression and response to treatment, ultimately contributing to better clinical outcomes for the elderly population affected by COVID-19.

These findings provide crucial insights for clinical decision-making. Specifically, the elevation of indices such as the systemic immune inflammation index and the derived neutrophil-to-lymphocyte ratio index in patients over 80 years old have been linked to higher risks of severe disease, ICU admission, and mortality. This information is especially valuable in the elderly population, who are already at increased risk due to higher baseline comorbidities as evidenced by their higher Charlson Comorbidity Index scores. The clinical utility of these findings lies in the potential for these biomarkers to serve as early indicators of poor prognosis, thereby guiding more aggressive or tailored therapeutic interventions. Moreover, the significant differences in laboratory markers such as neutrophil counts and AST levels between elderly patients and a younger control group underscore the necessity for age-specific clinical thresholds and management strategies.

### 4.2. Study Limitations

This study has several limitations. First, its retrospective design may introduce selection bias, and causality cannot be established. Second, the sample size, while adequate, may limit the generalizability of the findings. Third, we excluded patients with chronic liver disease, which may affect the applicability of the results to patients with hepatic conditions. Additionally, we did not assess dynamic changes in the indices over time, which may provide further prognostic information. Future prospective studies with larger sample sizes and diverse populations are needed to validate these findings and explore the utility of these indices over the course of the disease.

## 5. Conclusions

In conclusion, SII and dNLR are valuable prognostic biomarkers for predicting severe disease, ICU admission, and mortality in COVID-19 patients aged 80 years and above. Their use can aid clinicians in early identification of high-risk patients, allowing for timely interventions and optimized resource allocation. Incorporating these indices into clinical practice may enhance risk stratification and improve patient outcomes. Further research is warranted to validate these results and explore the mechanisms underlying the predictive capabilities of these biomarkers.

## Figures and Tables

**Table 1:** Demographic and clinical characteristics of study participants.

Variables	Elderly Patients (n = 138)	Control Patients (n = 215)	*p*
Age (years), mean ± SD	83.7 ± 3.1	52.5 ± 9.2	<0.001
Female sex, n (%)	80 (58%)	122 (57%)	0.816
BMI (kg/m²), mean ± SD	24.6 ± 3.9	26.8 ± 4.7	<0.001
Smoking history, n (%)	16 (12%)	58 (27%)	<0.001
Alcohol use, n (%)	12 (9%)	46 (21%)	<0.001
Vaccinated against COVID-19, n (%)	76 (55%)	132 (61%)	0.219
Charlson Comorbidity Index, mean ± SD	4.4 ± 1.3	2.0 ± 0.8	<0.001
Hypertension, n (%)	98 (71%)	82 (38%)	<0.001
Diabetes mellitus, n (%)	62 (45%)	42 (20%)	<0.001
Chronic kidney disease, n (%)	28 (20%)	11 (5%)	<0.001
CCI > 3	22 (16%)	15 (7%)	0.012
**Clinical severity of COVID-19 on admission, n (%)**			0.214
Mild	32 (23%)	66 (30%)	
Moderate	52 (38%)	81 (38%)	
Severe	54 (39%)	68 (32%)	
ICU admission, n (%)	36 (26%)	22 (10%)	<0.001
Mechanical ventilation, n (%)	24 (17%)	14 (7%)	<0.001
Mortality, n (%)	30 (22%)	12 (6%)	<0.001

BMI—body mass index; SD—standard deviation; ICU—intensive care unit; A CCI score higher than 3 is considered of high severity.

**Table 2:** Laboratory findings and hematological indices at admission.

Variables	Elderly Patients (n = 138)	Control Patients (n = 215)	*p*
Neutrophil count (×10⁹/L), mean ± SD	7.9 ± 2.6	5.5 ± 2.2	<0.001
Lymphocyte count (×10⁹/L), mean ± SD	0.9 ± 0.4	1.5 ± 0.5	<0.001
Platelet count (×10⁹/L), mean ± SD	178 ± 52	222 ± 64	<0.001
AST (U/L), mean ± SD	56 ± 21	38 ± 14	<0.001
Total leukocyte count (×10⁹/L), mean ± SD	9.6 ± 3.1	7.4 ± 2.6	<0.001
**Calculated Indices**			<0.001
Derived NLR (dNLR), mean ± SD	1.1 ± 0.4	0.8 ± 0.3	<0.001
ALRI, mean ± SD	62.2 ± 28.5	24.8 ± 11.5	<0.001
APRI, mean ± SD	0.79 ± 0.33	0.35 ± 0.14	<0.001
SII (×10⁹/L), mean ± SD	1325 ± 510	590 ± 240	<0.001

SD—standard deviation; dNLR—derived neutrophil-to-lymphocyte ratio; ALRI—aspartate-aminotransferase-to-lymphocyte ratio index; APRI—aspartate-aminotransferase-to-platelet ratio index; SII—systemic immune inflammation index.

**Table 3:** Severe disease prediction using hematological indices in elderly patients (>80 Years Old).

Indices	AUC	95% CI	*p*	Cutoff	Sensitivity (%)	Specificity (%)
dNLR	0.792	0.722–0.862	<0.001	1.1	80%	73%
ALRI	0.763	0.690–0.836	<0.001	46	76%	68%
APRI	0.729	0.652–0.806	<0.001	0.66	71%	64%
SII	0.857	0.795–0.919	<0.001	920	86%	78%

CI—confidence interval; AUC—area under curve; dNLR—derived neutrophil-to-lymphocyte ratio; ALRI—aspartate-aminotransferase-to-lymphocyte ratio index; APRI—aspartate-aminotransferase-to-platelet ratio index; SII—systemic immune inflammation index.

**Table 4:** ICU admission prediction using hematological indices in elderly patients (>80 years old).

Indices	AUC	95% CI	*p*	Cutoff	Sensitivity (%)	Specificity (%)
dNLR	0.805	0.734–0.876	<0.001	3.2	83%	76%
ALRI	0.778	0.708–0.840	<0.001	53	79%	70%
APRI	0.754	0.681–0.827	<0.001	0.74	75%	67%
SII	0.869	0.801–0.934	<0.001	966	88%	81%

CI—confidence interval; AUC—area under curve; dNLR—derived neutrophil-to-lymphocyte ratio; ALRI—aspartate-aminotransferase-to-lymphocyte ratio index; APRI—aspartate-aminotransferase-to-platelet ratio index; SII—systemic immune inflammation index.

**Table 5:** Cox regression analysis for predictors of mortality in elderly patients.

Variables	Hazard Ratio	95% CI	*p*
Age	1.04	0.98–1.10	0.18
Female sex	1.1	0.7–1.7	0.65
Charlson Comorbidity Index > 2	1.3	1.1–1.5	0.004
SII (>920 × 10⁹/L)	3.2	1.9–5.2	<0.001
dNLR (>1.1)	2.4	1.5–3.8	0.001
ALRI (>46)	1.2	0.8–1.9	0.29
APRI (>0.66)	1.1	0.7–1.7	0.51

dNLR—derived neutrophil-to-lymphocyte ratio; ALRI—aspartate-aminotransferase-to-lymphocyte ratio index; APRI—aspartate-aminotransferase-to-platelet ratio index; SII—systemic immune inflammation index.

## Data Availability

The data presented in this study are available on request from the corresponding author.
